# Impact of structured basic life-support course on nurses' cardiopulmonary resuscitation knowledge and skills: Experience of a paediatric department in low-resource country

**DOI:** 10.1016/j.afjem.2021.03.014

**Published:** 2021-07-22

**Authors:** Christian Umuhoza, Lei Chen, Juliette Unyuzumutima, Natalie McCall

**Affiliations:** a Paediatrics, University of Rwanda, Kigali City, Rwanda; b Paediatrics, Centre Hospitalier Universitaire de Kigali (CHUK), Kigali City, Rwanda; c Paediatrics, Yale University, New Haven, CT, United States of America

**Keywords:** Basic life support (BLS), Cardiopulmonary resuscitation (CPR), Education, Low-resource, Developing countries

## Abstract

**Introduction:**

The study aimed to assess the impact of a modified paediatric basic life support (BLS) training on paediatric nurses' knowledge and skills in the main tertiary level public hospital in Rwanda.

**Methods:**

A prospective, before-and-after educational intervention study was performed. Nurses working in the paediatric department at Centre Hospitalier Universitaire de Kigali (CHUK) were enrolled after consenting to the study. A modified BLS training was administered using didactic lectures, videos, case discussions, and simulations. Knowledge and skills were assessed before, immediately and six months after the training, using the American Heart Association (AHA) multiple-choice questions test and simulation scenarios. Ethical approval from the hospital's investigational review board was obtained before the start of the study.

**Results:**

Fifty-seven nurses working in paediatric department were included in the study, most with advanced nursing degrees. At baseline, only 3.5% scored above 80% on the knowledge test and none were able to perform high-quality one-rescuer CPR. Knowledge and high-quality one-rescuer CPR skills improved significantly immediately after the training, with 63.2% scoring above 80% and 63.2% capable of performing high-quality one-rescuer CPR (*p* < 0.01). Six months later, only 45.6% scored above 80% and 15.8% were capable of performing high-quality one-rescuer CPR (p < 0.01). Some skills, such as delivering breaths using bag-mask device, showed better retention.

**Conclusion:**

In the paediatric department of the main public tertiary care hospital in Rwanda, nurses' baseline knowledge and skills in providing BLS was poor but can increase with focused BLS training. Due to the decline in knowledge and skills over six months, the use of debriefing and focused trainings following resuscitation events and improved implementation of yearly departmental refresher courses are recommended.

## African relevance

• Successful effort to adapt the curriculum to locations with fewer resources.
• Generalisability of findings toward similar healthcare settings.

## Introduction

The majority of childhood deaths occur in low-resource countries, especially in Sub-Saharan Africa [1]. Rwanda has a decentralised healthcare system, where acutely ill children are referred up to higher levels of care [2]. As a consequence, hospitals such as Centre Universitaire Hospitalier de Kigali (CHUK), the largest tertiary hospital in Rwanda, have a higher burden of acutely ill children, with some of these children developing cardio-respiratory arrest and requiring resuscitation. The nursing staff is on the front-line and is most of the time called to perform such resuscitations.

Poor skills in life support procedures among health care providers are associated with increased morbidity and mortality among critically ill patients who experience cardiac arrest [3]. Nurses are usually the first to detect cardiac and/or respiratory arrest and the first to initiate BLS and further management which, if done properly, may ultimately lead to reduced mortality. Therefore, it is essential to have a well-trained nursing staff and fully equipped to perform efficient life support.

The literature reports poor knowledge and skill retention of cardiovascular resuscitation skills among healthcare providers, including doctors and nurses [4]. Studies have shown that training healthcare providers in cardiopulmonary resuscitation could lead to better outcomes [5,6].

The available evidence supports the need to train nurses in life support techniques [7,8]. Training using well-designed and systematic life support skills has benefits even in more seasoned nurses with experience in caring for patients with emergencies [9]. In the paediatric population, it was shown that training in cardiovascular resuscitation was associated with improved knowledge and skills immediately after training [10]. It was also reported that skills wane more quickly than knowledge [11]. Those findings are very concerning for healthcare workers caring for critically ill patients for whom resuscitation skills must be recalled immediately and performed with a high degree of accuracy [12]. Time to effective CPR is associated with outcome in inpatient cardiac arrests [13,14]. Since nurses are more likely to be present during inpatient paediatric arrests, especially in low-resource countries where physician density is lower, we believe it's important to teach and maintain CPR skills in nurses. Therefore refresher courses are an alternative to deal with the issue of retention of the learned skills, and many studies recommend multiple and frequent refresher courses for healthcare workers [15].

The objective of this hospital-based study was to assess whether a limited, low-cost training in BLS would lead to a sustainable increase in BLS knowledge and skills of paediatric nurses 6 months after the training.

The reporting of this study is done according to the Guidelines for Reporting Evidence-based practice Educational interventions and Teaching (GREET).

## Methods

### Study design

This is prospective, before and after an educational intervention study of a group of nurses.

### Study setting

The study was conducted in CHUK, a hospital that receives paediatric and adult patients, and provides medical, surgical and paramedical services, and is a tertiary care hospital serving as a referral centre for two-thirds of the district hospitals in the country.

The hospital has developed policies on CPR and training of staff in life support. The policies state that all healthcare providers should be trained in BLS, with refresher courses every year. However, the implementation of these policies is still challenging.

The paediatric department has different units, including one ambulatory unit, hospitalisations units, one emergency unit, one paediatric high dependency unit, and one 3-beds paediatric intensive care unit. The paediatric department receives >300 paediatric patients per month, and some of these patients are critically ill; cardio-respiratory failure and arrest are common, with an average of 7 to 10 CPR per week. There are limited human resources, shortages of medications, equipment and supplies, insufficient intensive care beds, invasive ventilation and monitoring capabilities resulting in limited post-cardiac arrest monitoring and support, and limited long-term care of patients who have suffered a cardiac arrest; therefore, the outcomes for children who have suffered a cardiac arrest are poor. The mortality rate in paediatric department at CHUK is among the highest in the country, and a recent article reported a mortality as high as 50% among children admitted in the paediatric ICU [16].

Nurses working in paediatric feel that mastering the appropriate CPR skills will positively impact the paediatric patients' outcomes for the better.

### Study participants

There were 80 nurses in the Department of Paediatrics at CHUK at the time of the study. All nurses were eligible to participate in the study and were enrolled after providing a written consent to the study. The baseline experience in life support for nurses working in all paediatric units was variable; the majority had a poor baseline training experience in BLS or other related life support training programs. Nurses who consented to participate in the study were enrolled and randomly divided into groups of 10 people to attend the training according to their availability.

### Education intervention

The principal investigators designed a 4-hour Basic Life Support training course, specifically adapted for nurses in a resource-constrained environment. The hospital has a simulation centre, and that centre was used for the training sessions, which included the skill teaching sessions, lectures on the theoretical aspects of BLS, and final assessment. The principal investigator was the sole instructor for the course. The investigator is a certified trainer in many basic and advanced paediatric life support programs. The learning objective of the course was to provide the nurses with the ability to recognise life-threatening paediatric emergencies such as cardiac or respiratory arrest and the skills to provide CPR. The training included lectures, case discussions, demonstrations, and simulations. The course covered most of the content of the 2010 American Heart Association (AHA) BLS course. The theoretical aspect of the training was delivered through an interactive presentation given in French and Kinyarwanda, group discussion as well as videos from the 2010 AHA Guidelines for Cardiopulmonary Resuscitation and Emergency Cardiovascular Care [17]. The AHA BLS course content was adapted by removing the practical aspect of using an AED, due to the lack of this device in the facility. The AHA was not involved in the adaptation of the BLS curriculum. The adapted curriculum was reviewed by 2 specialists to increase content validity. The practical aspect of the course was provided through 2-hour interactive skill teaching sessions using manikins (BLS manikins and Baby Anne™ Infant manikin, Laerdal). One and two-person infant, child and adult resuscitations skills using CAB approach were covered. Eight training sessions were conducted between August and December 2017. There were no self-directed learning activities and the participants were not provided with the manual or course materials. All teaching was face-to-face and the ratio of student to the instructor was between 5:1 and 10:1. There were enough opportunities for individuals to practice skills. There were no modifications to the educational strategies during the study.

### Variables and data sources/measurements

The main outcome of interest was the knowledge and skills in BLS before and after the intervention. To assess pre- and post-course knowledge, a self-administered semi-structured questionnaire from the BLS curriculum was used. Questions included general knowledge in BLS, the indications of BLS, appropriate stabilisation of airways, breathing and circulatory support during resuscitation, according to the 2010 AHA guidelines. To assess pre- and post-course BLS skills, a one-person infant resuscitation scenario with cardio-respiratory arrest on a Baby Anne™ manikin was used. Skills were measured using the AHA Infant CPR Checklist tool [18]. The knowledge and skill assessments were performed before and immediately after the participants underwent the training, and again 6 months later. Remediation opportunities were offered to the participants whose skills and/or knowledge were determined to be inadequate (as defined by obtaining a score <80% on the knowledge test or by failure to perform high-quality CPR). This remediation was given right after taking the test. High-quality CPR was defined as per the 2010 AHA guidelines (compression started within 10 s after recognising a heart arrest, push hard, push fast, allowing complete chest recoil). Minimising interruptions to <10 s, giving effective breaths and avoiding excessive ventilation.

### Statistical methods

Data were entered using Epidata 3.2. and analysed through STATA (StataCorp. *Stata Statistical Software*, *version 13*. College Station, TX). Descriptive statistics i.e. percentage, mean, mode, median, interquartile range, and standard deviation were used to report findings of pre-test and pos*t*-test, with box plots used to report differences between groups. For intergroup comparison, paired t-test was used for paired numerical data, Chi-square and Fischer's exact test for independent categorical data and the McNemar test was used for paired ordinal categorical data. A statistical significance was considered if the *p*-value was <0.05.

### Ethical considerations

Ethical clearance from the Ethical committee at CHUK was obtained before the data collection (CHUK Ref EC/CHUK/388/2016). Informed consent was obtained from all the participants. Confidentiality was maintained by coding participant identifiers and by keeping the filled questionnaires in a safe and locked area only accessible by the principal investigator.

## Results

### Participants

Eighty nurses were working in the Paediatrics Department at the beginning of the study. Sixty-three were available and were enrolled. Six left CHUK before the end of the study. Fifty-seven subjects completed the follow-up and were included in the final analysis. We only considered those who were able to complete the follow up. The participant characteristics are shown in Table 1.

**Table 1:** Participants related characteristics.

Characteristics	Frequencies (N = 57)	Percentages
Age of the nurses, [median 37 (32,41) years]^a^		
≤35 years	26	(45.6%)
>35 years	31	(54.4%)
Gender		
Female	53	(93%)
Male	4	(7%)
Last CPR training experience, [median 6 (6,12) months]^a^		
≤6 months	38	(66.7%)
>6 months	19	(33.3%)
Level of training		
Secondary level (A2)	4	(7%)
Advanced diploma in nursing (A1)	46	(80.7%)
Bachelor degree (A0)	5	(9.8%)
Masters	2	(3.5%)
Years of practice		
≤5 years	13	(22.8%)
6 to 10 years	24	(42.1%)
>10 years	20	(35.1%)

a Median reported (no normality in distribution of data).

### Outcome

**Fig. 1:**
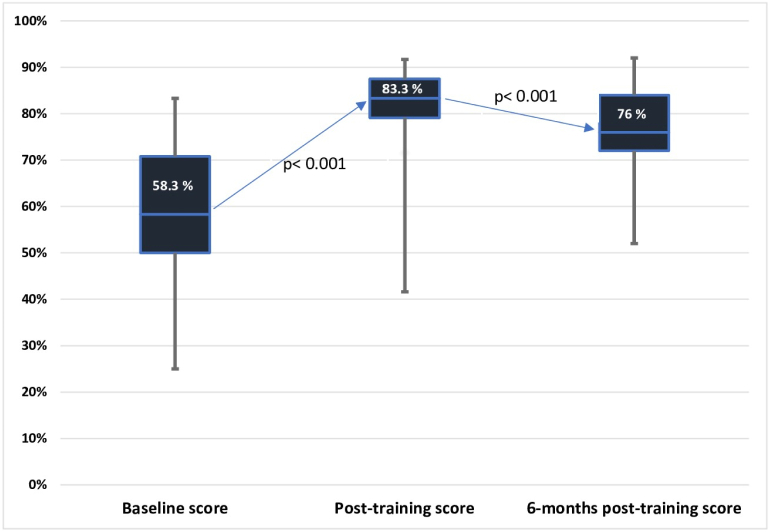
Knowledge acquisition.

Pre-intervention, post-intervention and 6 months' follow-up assessment results of the knowledge and skill assessments are shown in Fig. 1, Table 2, Table 3. There was an increase in mean knowledge scores from 58.3% before the training to 83.3% after the training, which decreased to 76% at 6 months after the training. There was a statistically significant increase in knowledge (Fig. 1) and high-quality CPR skills (Tables 2 and 3) from before to after the training (*p* < 0.01) and a statistically significant decline in skill 6 months after the training (p < 0.01) compared to the immediately after-training period.

**Table 2:** Participants' knowledge and skills related characteristics.

Items assessed	Baseline assessment score	Post-intervention assessment score	6 months post-assessment scores
	N (57)	N (57)	N (57)
Knowledge questionnaire			
≤60 points	30 (53%)	1 (2%)	4 (7%)
60–80 points	25 (44%)	20 (35%)	33 (58%)
>80 points	2 (3%)	36 (63%)	20 (35%)

High quality CPR for 1 rescuer			
Good performance	0 (0%)	36 (63%)	9 (16%)
Unsatisfactory performance	57 (100%)	21 (37%)	48 (84%)

Effective delivering of breaths with bag-mask device			
Good performance	14 (24.6%)	53 (93%)	55 (97%)
Unsatisfactory performance	43 (75.4%)	4 (7%)	2 (4%)

Effective chest compressions if 2 rescuers			
Good performance	16 (28%)	49 (86%)	36 (63%)
Unsatisfactory performance	41 (72%)	8 (14%)	21(37%)

**Table 3:** Skills acquisition before training, just after training and 6 months after the BLS training.

Variables	Before training (N = 57)	Immediately after training (N = 57)	6-month post-training (N = 57)	Comparison of before and after training^a^	Comparison of after training and 6 months later^a^	Comparison of before training and 6 months later^a^
Skill correctly performed	n (%)	n (%)	n (%)	P value	P value	P value
Assessment	35 (61.4%)	57 (100%)	52 (91.2%)	<0.001	0.06	<0.001
Call for help/AED	27 (47.4%)	52 (91.2%)	41 (71.9%)	<0.001	0.007	0.008
Check for pulse	20 (35%)	54 (94.7%)	38 (66.7%)	<0.001	<0.001	0.001
Correct compressions	16 (28%)	51 (89.5%)	37 (64.9%)	<0.001	0.006	<0.001
Adequate rate	6 (10.5%)	49 (86%)	12 (21.1%)	<0.001	<0.001	0.14
Adequate depth	39 (68.4%)	55 (96.5%)	48 (84.2%)	<0.001	0.06	0.06
Allows complete chest recoil	33 (57.9%)	57 (100%)	50 (87.7%)	<0.001	0.01	<0.001
Minimise interruptions	17 (29.8%)	48 (84.2%)	44 (77.2%)	<0.001	0.48	<0.001
High quality CPR for 1 rescuer	0 (0%)	36 (63.2%)	9 (15.8%)	<0.001	<0.001	0.003
Effective delivery of breaths with bag-mask device	14 (24.5%)	53 (93%)	55 (96.5%)	<0.001	0.62	<0.001
Effective chest compressions if 2 rescuers	16 (28.1%)	49 (86%)	36 (63.2%)	<0.001	0.007	<0.001

a McNemar's test for comparison of the paired ordinal categorical data.

There was a statistically significant increase in the components of skills necessary for a one-person infant resuscitation right after training compared to baseline. However, there was some variation in skill retention, with a significant decline in skills such as calling for help, checking for pulse, correct compression technique, adequate rate, and allowing complete chest recoil.

However, despite the decline in these skills, we found that all components of skills except for providing chest compression with an adequate rate were significantly improved 6 months after training compared to baseline (Table 3).

A bivariate analysis was undertaken to assess whether the age of the nurses, time of most recent CPR training experience, years of practice or ward assignment was associated with baseline knowledge found that there was no statistically significant association (p-value >0.05).

## Discussion

Previous research on BLS in paediatric nurses is often conducted in settings with significant healthcare resources. To our knowledge, this is the first research project conducted in Rwanda to evaluate the BLS knowledge and skills of nurses in a paediatric Department. CHUK is the main public referral hospital in Rwanda, a Sub-Saharan African country. Findings from our study may have implications for a wide variety of settings with similar healthcare, education, and infrastructure constraints. We identified deficiencies at the baseline, designed and adapted a well-established BLS training package from resources freely available to the public, and demonstrated significant improvement after implementing this low-cost training. Finally, after six months we observed retention of certain aspects of the training and attrition of others.

### Pre-test knowledge of cardiopulmonary resuscitation

This study showed a significant baseline deficiency in BLS knowledge and skills among nurses working in paediatrics at CHUK, a tertiary hospital in a low-resource setting in Sub-Saharan Africa.

The pre-intervention knowledge median score of 58.3% pointed to deficiencies in knowledge. Some deficiencies identified included knowledge of correct compression-ventilation ratio, compression rate, BLS steps, chain of survival and the indication for using an automated external defibrillator (AED). Skills such as providing effective ventilation using the bag-valve-mask were deemed deficient. These steps are essential during CPR and studies reported that incorrect steps in initiating CPR process can lead to poor outcomes with reduced chances of survival after a cardiac arrest [19]. These findings could be explained by the fact that 33.3% of the trained nurses received their last formal CPR training >6 months before the time of our study (Table 1) and that, previously to this study, the policy of yearly training in CPR was not widely implemented in our hospital. Also, during resuscitations on patients and because of staffing issues, nurses may not be receiving feedback as debriefing sessions are rarely if ever done. Previous research showed that retention of BLS knowledge decrease over time among trained healthcare providers and nursing students [20]. Nurses' knowledge levels have been reported to be also influenced by factors such as their motivation, attitude and willingness to learn BLS or participate in drills [21].

### Pre-test skills of cardiopulmonary resuscitation

The cardiopulmonary resuscitation skills of the registered nurses working in paediatrics were poor during the pre-intervention part of our study. Identified deficits include essential skills such as checking the scene safety, appropriately checking the pulse. Very few of them were able to activate the emergency response system and none of them was able to give high-quality CPR. The compression rates and the use of the bag-valve-mask were not accurate for the majority of nurses (Table 3). Poor skills in delivering high-quality CPR could result in poor outcome in paediatric victims of a cardiac arrest [22]. The literature reports that inaccurate skills in providing ventilation during a cardiac arrest event by a bag-valve-mask could result in hyperventilating the victim, which could lead to decreased coronary perfusion pressures and survival rates among the cardiac arrest victims [23].

Poor training experience in cardiopulmonary resuscitation could be the explanation of why the majority of nurses performed poorly at baseline. The traditional role of the nurse in resuscitation in Rwanda is generally passive, with paediatric residents and consultants typically leading and performing the entire resuscitation event. Such limited exposure to resuscitation has been reported as one cause of poor cardiopulmonary skills and knowledge among nursing healthcare professionals [24]. Paediatric residents and consultants in CHUK are generally more skilled in CPR and most of them receive regular training and refresher courses in paediatric CPR.

Although 66.7% of the nurses had completed CPR training within 6 months of entering the study, they had very poor knowledge and skills. This may be related to the previous BLS curriculums which were provided at the hospital level, and were targeting all healthcare providers, regardless of their different departments or units. CPR trainings provided at the departmental level may be more accurate for knowledge and skills retention. This needs further studies.

### Improvement after initial training

This study showed that nurses can achieve significant improvements in both their knowledge and skills after training in BLS, using an adapted BLS curriculum. Similar findings were reported previously [25].

### Retention at 6 months

Our study showed that the retention of both knowledge and skills decreased at 6 months after the training but remained significantly improved compared to baseline. Specific skills such as assessing the pulse, performing correct chest compressions with the correct rate for a single rescuer, and performing high-quality CPR declined significantly over the 6 months. Many researchers highlighted similar findings with declines in both knowledge and skills in BLS starting 3 months after the training [11]. Interestingly some skills were retained. By 6 months, post-training skills were in general better, compared with baseline, for most items except for the adequate rate and depth of chest compressions. Retention of these skills may be due to ongoing exposure to clinical situations where such skills are needed and used. Skills for bag-mask ventilation were retained relatively well, and one explanation possible would be that during CPR, nurses are generally the ones to initiate and perform bag-mask ventilations. Another possible explanation could relate to the positive experience of very successful response to bagging for respiratory arrest and apnoea. On the other hand, the fact that most chest compressions are generally performed by residents and consultants could explain why chest compression skills are poorly retained; therefore, infrequently used skills may tend to wane and deteriorate over time. In addition, it has been reported that correct compression rate and depth tend to be poorly retained for prolonged periods [26].

In our study, years of practice did not affect final performance. It has previously been observed that while work experience may increase the confidence level of individual nurses, there is no correlation between years of work experience and competencies in the performance of CPR [27]. The unit or place of work was not associated with any difference in the performance of CPR among nurses. Other authors have conversely reported that nurses working in high-risk areas such as the Intensive Care Unit (ICU) and nurses working continuously in close contact with patients are more motivated to maintain their competence in CPR than other health care professionals [28]. However, the level of acuity of patients referred to our hospital is high in all units of the Department. Even if we have a paediatric ICU unit, the bed capacity is very limited and most of the critically ill paediatric patients will be managed outside paediatric ICU; therefore, nurses working in paediatrics are more likely to be exposed to more critically ill patients and more CPR in their different paediatric units. Age also was not associated with the final score. Similar results were reported in a study conducted in Kenya, where factors such as age, gender, and work experience did not have a significant association with the level of CPR performance among nurses [29].

Our study has several limitations. First, the study was only conducted in a paediatric department of a tertiary hospital in Rwanda, and only nurses were involved in the study. The results may not be generalised to the nurses working in other healthcare facilities where baseline characteristics and on-going exposures are different. Skills were evaluated using simulated scenarios which may not reflect the performance during the actual patient encounter [30].

Changes in the outcome of interest (which is an improvement in knowledge and skills in paediatric basic life support techniques) were presumed to be the result of the intervention but it is possible that other ongoing training and exposure to resuscitations could have contributed to acquisition and retention of knowledge and skills.

And finally, the course was led by a single instructor, which means that the impact on score performance may not be able to be generalised more widely.

We adapted the curriculum to suit the resources available in our setting. For example, we did not fully train the nurses on the AED, because those devices weren't available in our hospital. Despite the differences from the standard AHA course, we were able to effectively demonstrate an improvement in skills and knowledge not only after the study but retention six months later. This represents a successful effort to adapt a curriculum to locations with fewer resources.

## Conclusion

Our study showed that there is a significant improvement in knowledge and skills after a brief training in BLS among paediatric nurses in a tertiary care hospital in urban Rwanda. Some knowledge and skills were retained after six months. Due to declines in knowledge and skills, we recommend the use of debriefing and focused trainings following resuscitations episodes, and improved implementation strategies of yearly refresher courses at the departmental level. Finally, further studies should also focus on examining the patient outcomes following training in BLS such as this one.

## Dissemination of results

Results from this study were shared with staff members at CHUK through an informal presentation.

## Authors' contributions

Authors contributed as follow to the conception or design of the work; the acquisition, analysis, or interpretation of data for the work; and drafting the work or revising it critically for important intellectual content: CU contributed 50%; NMC 25%; LC 20%, and JU 5%. All authors approved the version to be published and agreed to be accountable for all aspects of the work.

## Declaration of competing interest

The principal investigator is also employed by CHUK. The authors declared no further conflict of interest.
